# Extraordinary Case, in Which the Arm Was Torn off at the Shoulder-joint, by Machinery

**Published:** 1843-10

**Authors:** Jas. S. Cooper

**Affiliations:** British Guiana, South America


*Extraordinary Case, in which the arm was torn off at the Shoulder-joint, by machinery, communicated to Prof. Willard Parker, by* Jas. S. Cooper, M.D., *of British Guiana, South America.*—I was called to a neighboring plantation to see

Daniel, aged about 7 years, son of one of the laborers. On arriving, I found a case of a very serious nature. The boy, while playing about the sugar-mill of the estate, had been caught in some of the machinery, and his left arm was torn off, bringing with it about two-thirds of the scapula. The deltoid muscle was carried away entire, and the trapezius was torn from its attachment to the clavicle, the naked end of which bone was protruding through the wound. Notwithstanding the very large amount of surface exposed, there was not the slightest hemorrhage, although reaction had fairly taken place.

With the assistance of Dr. Walker, the medical attendant of the estate, I proceeded to dissect out the remainder of the scapula, (the inferior and posterior portion). A little bleeding ensued from the scapular arteries, but it soon ceased on pressure being made. Then, with a small saw, I removed the external third of the clavicle. As there was no necessity for tying a single vessel during the whole operation, the wound was now closed with sutures and adhesive straps; and the dressing was finished, by applying a compress and roller around the body. The patient bore the operation well, and soon after fell asleep.

Dec. 16.—Considerable febrile action—pulse 120—tongue furred—wound rather painful. Ordered a slight purgative and left him.

20th.—Every thing has gone on well up to this day; the wound was dressed; about half of it had united by the first intention, and the remainder is suppurating kindly. There is no fever; and the patient is every way comfortable, sitting up in bed, he having taken a full meal. The sutures were removed; and from this time onward he recovered rapidly.

*New York Journal of Medicine.*

